# Sjögren’s syndrome presents with recurrent spontaneous pneumothorax

**DOI:** 10.1515/rir-2026-0023

**Published:** 2026-07-13

**Authors:** Yuyan Chao, Yiyu Zou, Rong Li, Lidan Zhao

**Affiliations:** Department of Rheumatology and Clinical Immunology, National Clinical Research Center for Dermatologic and Immunologic Diseases (NCRC-DID), The Ministry of Education Key Laboratory, Peking Union Medical College Hospital, Chinese Academy of Medical Sciences, Peking Union Medical College, Beijing, China; Department of Rheumatology and Immunology, Longkou People’s Hospital, Shandong Province, Longkou, China

Dear Editor,

Primary Sjögren’s syndrome (pSS) is a chronic autoimmune disorder characterized by lymphocytic infiltration and destruction of exocrine glands, resulting in xerostomia, xerophthalmia, and extraglandular manifestations.^[1]^ Pulmonary complications implicate up to 20% pSS patients and are one of the serious types of visceral involvement. Lymphoid interstitial pneumonia (LIP) with thin-walled cysts is the most common, and is often associated with lymphoproliferative disorders.^[2,3]^ Whereas pneumothorax is extremely rare and seldom represents the initial presentation. Consequently, therapeutic experience in such cases remains limited and autoimmune background might be neglected. Here, we report a young woman whose first and predominant manifestation of pSS was recurrent spontaneous pneumothorax associated with extensive pulmonary bullae, and we also reviewed similar cases in literatures, aiming to highlight this atypical presentation and its therapeutic challenges.

A 29-year-old woman first presented in March 2021 with sudden chest pain and dyspnea. A chest X-ray revealed a right-sided pneumothorax and closed thoracic drainage was performed with symptoms relieved. Nine months later, she experienced another episode of spontaneous pneumothorax on the left side. Laboratory examinations showed slight leukopenia (WBC 3.01×109/L), positive ANA (1:160), positive anti-SSA and anti-Ro52 antibodies. Coombs test was positive along with elevated IgG levels (20.23 g/L). Salivary gland scintigraphy indicated severe bilateral parotid gland dysfunction. Sjogren syndrome was diagnosed despite of the absence of apparent sicca symptoms or other complaints such as parotid gland swelling, oral ulcers, or arthralgia.

She was referred to our center in January 2022. High-resolution chest computed tomography (HRCT) showed left-sided hydropneumothorax, minor right-sided pneumothorax, bilateral pulmonary bullae and emphysema, left lower lobe patchy opacities with possible atelectasis (Figure. 1A), suggesting possible LIP. Treatment of prednisone (40 mg/d), mycophenolate mofetil (MMF)(0.75 g twice daily), and hydroxychloroquine (HCQ)(0.2 g twice daily) was initiated, but pneumothorax reoccurred and aggravated during steroid tapered to 35 mg/d, necessitating another closed thoracic drainage in March 2022. Prednisone was gradually reduced to 10 mg/d as her condition stabilized, but imaging showed extensive pulmonary bullae.

**Figure 1:**
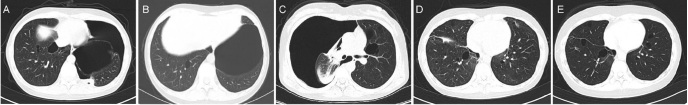
Radiographic and computed tomography (CT) findings of recurrent spontaneous pneumothorax in the patient with Sjögren’s syndrome. A, High-resolution CT in January 2022 showing left-sided hydropneumothorax, minor right-sided pneumothorax, bilateral pulmonary bullae and emphysema, and patchy opacities in the left lower lobe with possible atelectasis. B, CT scan in July 2022 after thoracoscopic wedge resection and pleurodesis, showing residual pulmonary bullae. C, CT scan in March 2024 revealing recurrent right-sided pneumothorax and multiple bullae, necessitating repeat surgical intervention. D, CT scan in July 2024, one month after initiation of tofacitinib therapy, demonstrating resolution of pneumothorax with persistent but stable pulmonary bullae. E, CT scan in July 2025, after one year of tofacitinib treatment, revealing multiple bullae but no pneumothorax.

In July 2022, thoracoscopic wedge resection and pleurodesis were performed, yet residual bullae still can be observed on chest CT (Figure 1B). Surgical histopathology confirmed pulmonary bullae but showed no evidence of smooth muscle proliferation or Langerhans cell infiltration. What’s more, cysts on chest imaging were not diffuse and uniform like those typical in Lymphangioleiomyomatosis (LAM) nor the irregular, nodular upper-lobe scattered that typical of Pulmonary Langerhans Cell Histiocytosis (PLCH), thus excluding differential diagnosis of LAM and PLCH.

In March 2024, the patient developed recurrent right-sided pneumothorax despite ongoing immunosuppressive therapy (Figure 1C). A second thoracoscopic resection of bullae and pleurodesis, and tofacitinib (5 mg twice daily) was added to her treatment regimen. Over one year of follow-up, no recurrence was observed. Follow-up CT images at one month and one year are shown in Figure 1D–E. Corticosteroids were tapered to 2.5 mg/d, and blood counts and inflammatory markers remained stable (laboratory data from January 18, 2022, to June 13, 2025, are shown in Supplementary Table S1). Figure 2 provides a timeline detailing the development of the disease and the treatments received.

**Figure 2:**
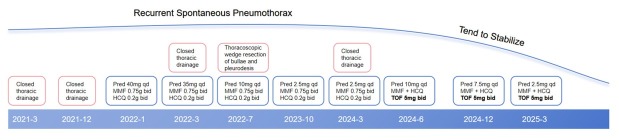
A timeline showing the development of the disease and the treatments received. Pred, prednisone; MMF, mycophenolate mofetil; HCQ, hydroxychloroquine; TOF, tofacitinib.

Pneumothorax in pSS is exceedingly rare and is thought to arise secondary to cystic lung disease caused by chronic lymphocytic infiltration of the small airways and peribronchiolar regions. This inflammatory process leads to bronchiolar narrowing or obstruction, resulting in air trapping, distal hyperinflation, and subsequent formation of cystic lesions or bullae. Over time, destruction of alveolar septa and remodeling of the airway architecture further contribute to the fragility of the affected lung parenchyma. Spontaneous pneumothorax may occur as a secondary event following rupture of these thin-walled cysts or bullae, particularly in the setting of ongoing inflammation or increased intrathoracic pressure. Given the rarity of this complication, standardized therapeutic approaches are lacking. By literature searching, only three cases of pneumothorax in pSS have been reported (Supplementary Table S2).^[4, 5, 6]^ Two patients received glucocorticoids and immunosuppressants, and all underwent surgical intervention. One patient died of respiratory failure, while the others achieved clinical stability.

In our patient, satisfactory disease control was achieved with combined therapy including corticosteroids, mycophenolate mofetil, hydroxychloroquine, and tofacitinib along with thoracoscopic bullae resection and pleurodesis. Studies have shown that STAT4 haplotype variants increase the risk of pSS.^[7,8]^ STAT4 is a critical intracellular signaling molecule in the interleukin (IL)-12 and IL-23 pathways, promoting the differentiation of Th1 and Th17 cells. Tofacitinib, an oral small-molecule Janus kinase (JAK) inhibitor, targets the JAK family, which activate STATs *via* autophosphorylation. Barrera *et al*. reported that tofacitinib could reduce epithelial inflammation in labial salivary glands of pSS patients and prevent the reduction of autophagy.^[9]^ A prospective clinical trial investigating the efficacy of tofacitinib in pSS-related interstitial lung disease is currently underway in China,^[10]^ and hoping in near future, substantial advancement on the efficacy of JAK inhibitors in lung involvement of pSS patients could be achieved. This report highlights the importance of recognizing atypical pulmonary involvement in pSS and reminds clinicians of screening autoimmunity in patients with unexplained pneumothorax, also this case may provide insights into potentially beneficial therapeutic management.

## Supplementary information

*Supplementary materials are only available at the official site of the journal (www.rir-journal.com*).

## Supplementary Material

Supplementary Material Details
