# Supplementary material for: Sjögren’s syndrome presents with recurrent spontaneous pneumothorax
Source: Rheumatol Immunol Res. 2026 Jul 13;7(2):131–3. doi: 10.1515/rir-2026-0023 (PMC13358942; doi:10.1515/rir-2026-0023)
Supplement: Supplementary file 1 — Supplementary Material Details [file rir-2026-0023_sm.pdf]

Supplementary Materials

Supplementary Table 1: Follow-up Laboratory data of the patient

	**2022/1/18 (baseline)**	**2023/10/24 (after treatment)**	**2024/9/3 (add tofacitinib)**	**2025/6/13**	**value**
WBC	3.01	2.62	4.01	4.77	3.5-9.5 $\times 10^9$/L
C3	1.121	1.021	0.992	1.121	0.73-1.46 g/L
C4	0.164	0.175	0.13	0.153	0.1-0.4 g/L
IgG	20.23	12.94	12.68	11.07	7-17 g/L
IgA	1.47	1.09	1.29	1.31	0.7-4 g/L
IgM	0.51	0.39	0.43	0.49	0.4-2.3 g/L
hs-CRP	0.28	0.19	0.13	0.09	<8 mg/L
ESR	14	7	2	3	0-15 mm/h
ANA	(+) S1: 640	(+) S1: 320	(+) S1: 320	(+) S1: 320	<1: 80

WBC, white blood cell; C3, complement 3; C4, complement 4; Ig, immunoglobulin; hs-CRP, hypersensitive C-reactive protein; ESR, erythrocyte sedimentation rate; ANA, antinuclear antibody.

Supplementary Table 2. Summary of case reports of Sjögren's syndrome complicated by pneumothorax

	Sex	Age	Clinical manifestations	Laboratory Examination	Imaging Examination	Treatment	Outcome	Reference
1	Female	75	Mild ocular dryness; Xerostomia; Right-sided pneumothorax	ANA negative Anti-SSA positive ESR: 39 mm/h	Multiple bilateral thin-walled cysts	Closed thoracic drainage; Thoracoscopic surgery	Clinical improvement	[5]
2	Female	57	Recurrent right-sided pneumothorax; Pneumonia-induced respiratory failure	---	Bilateral pulmonary bullae	Closed thoracic drainage; Prednisone	The patient developed respiratory failure and died.	[6]
3	Female	60	Dyspnea; Xerostomia; Ocular dryness; Tongue ulcers; Alopecia; Arthralgia; Recurrent left-sided pneumothorax	ANA positive Anti-SSAnegative Anti-SSB negative ESR: 65 mm/h	Left-sided pneumothorax	Closed thoracic drainage; Prednisone and Azathioprine	Clinical improvement	[7]
4	Female	29	Recurrent spontaneous pneumothorax	ANA positive Anti-SSA positive Serum IgG: 20.23 g/L ESR: 14 mm/h	Multiple pulmonary bullae	Closed thoracic drainage; Prednisone, Mycophenolate mofetil, Hydroxychloroquine and Tofacitinib	In a stable condition	Our case
